# Protective potential of bempedoic acid as an AMPK activator in tamoxifen-induced steatohepatitis in rats

**DOI:** 10.1007/s00210-025-04047-5

**Published:** 2025-04-10

**Authors:** Mona Mosaad, Elsayed K. El-Sayed, Engy M. El Morsy

**Affiliations:** 1 Dermatology Hospital, Damietta, Egypt; 2 Department of Pharmacology and Toxicology, Faculty of Pharmacy, Helwan University, Cairo, 11795 Egypt

**Keywords:** Tamoxifen, MASLD, Steatohepatitis, Bempedoic acid, AMPK

## Abstract

Despite the beneficial therapeutic effects of tamoxifen (TAMX) against breast cancer, long-term treatment with TAMX enhances the development of metabolic dysfunction-associated steatotic liver disease (MASLD), including steatohepatitis. Bempedoic acid (BA) is a recently approved lipid-lowering drug for dyslipidemia. Our study aimed to examine the potential impact of BA against TAMX-induced steatohepatitis in rats and underline the possible molecular pathways involved. Twenty-four adult female rats were allocated into 4 groups (*n* = 6): Control, TAMX (45 mg/kg), BA (15 mg/kg), and BA (30 mg/kg) groups. BA was given orally by gavage for 15 consecutive days. TAMX was administered to all rats except the normal control rats. Co**-**treatment of BA with TAMX showed well-organized structures of the hepatocytes in the histopathological sections. BA significantly improved liver function and lipid profile, demonstrating dose-dependent hepatoprotective effects. Mechanistically, BA significantly decreased oxidative stress by decreasing the level of malondialdehyde (MDA) and increasing the level of superoxide dismutase (SOD), significantly reduced inflammatory cytokines such as nuclear factor-kappa B (NF-κB/p65) and tumor necrosis factor-alpha (TNF-α), significantly inhibited lipogenesis by decreasing sterol regulatory element binding protein 1c (SREBP-1c), fatty acid synthetase (FAS), acetyl-coenzyme A carboxylases (ACC), ATP-citrate lyase (ACL), and significantly promoted fatty acid oxidation be enhancing carnitine palmitoyl transferase 1 significantly (CPT-1) through AMP-activated protein kinase (AMPK) activation. These findings suggest BA as a potential adjunct therapy for TAMX-induced metabolic dysfunction-associated steatohepatitis (MASH).

**Supplementary Information:**

The online version contains supplementary material available at 10.1007/s00210-025-04047-5.

## Introduction

Tamoxifen (TAMX) is an anti-estrogen drug commonly used as a therapeutic agent for the management of hormone-sensitive breast cancer, particularly for postmenopausal women (Chang et al. 2018). Deep vein thrombosis, vaginal bleeding, and cancer in the endometrium are adverse effects of long-term treatment with TAMX; however, metabolic dysfunction-associated steatotic liver disease (MASLD) is the greatest significant side effect linked with its use (Li et al. 2022).

Metabolic dysfunction-associated steatohepatitis (MASH) is an inflammatory subtype of MASLD, where the accumulation of fats in hepatocytes causes liver inflammation, damage of the hepatocytes, fibrosis, cirrhosis, and even hepatocellular carcinoma (Cuadrado et al. 2005). Hepatic fat accumulation is mainly caused by an imbalance between the deposition of fats and their clearance. This imbalance is attributed to increased hepatic fat production through increasing de novo free fatty acids (FFA) synthesis, declined FFA β-oxidation, and decreased output of triglyceride (TG) (Rodríguez et al. 2020). This results in steatosis and enhances the vulnerability to injury caused by lipotoxicity, mitochondrial dysfunction, cellular stress, inflammation, and induction of several cellular signaling pathways thought to be required to progress to liver fibrosis and failure (Wang et al. 2024).

TAMX is implicated in the pathogenesis of fatty liver via several mechanisms. It was documented that TMAX enhances lipid formation via upregulating the sterol regulatory element binding protein 1c (SREBP-1c), ATP-citrate lyase (ACL), acetyl-coenzyme A carboxylases (ACC), and fatty acid synthetase (FAS) (Zhao et al. 2014). Moreover, TAMX suppresses the oxidation of fatty acid by reducing carnitine palmitoyl transferase 1 (CPT-1), the enzyme initiating the first step for transportation of FFA into the mitochondria to be oxidized, halting the fat accumulation (Gudbrandsen et al. 2006).

In addition, TAMX has a detrimental effect on the liver tissues via activation of oxidative stress and inflammatory reactions. It is well-documented that TAMX enhances the overproduction of reactive oxygen species (ROS) and increases lipid peroxidation (Hassanein et al. 2018). Higher levels of ROS trigger the inflammatory response via activation of nuclear factor-kappa B (NF-κB) and inflammatory mediators, including tumor necrosis factor-alpha (TNF-α), producing liver damage (Kamel and Elariny 2023).

Bempedoic acid (BA) is a novel FDA-approved lipid-lowering drug used to manage dyslipidemia (Biolo et al. 2022). The mechanism of action of BA is the inhibition of ACL enzyme that acts upstream of HMG-CoA reductase (Nissen et al. 2023). In addition, BA possesses an anti-inflammatory effect via upregulation of the AMP-activated protein kinase (AMPK) (Biolo et al. 2022; Ahmed et al. 2023). BA is a prodrug that is mainly activated in the liver and remains unmetabolized in peripheral tissues like skeletal muscle, thereby lowering the risk of muscle injury, a common side effect of statins (Nissen et al. 2023). Recent studies suggest that AMPK activation can mitigate steatohepatitis through enhanced mitochondrial function and lipid homeostasis (Hammad et al. 2023; Bekheit et al. 2025).

Accordingly, due to the prolonged treatment duration and the pressing need for TAMX in breast cancer, as well as the promising hypolipidemic and anti-inflammatory effects of BA, our study aimed to explore the yet unexplored potential of BA in mitigating TAMX-induced steatohepatitis in rats. We also sought to examine the potential mechanisms underlying its protective action, thereby addressing the gap in understanding BA's role in TAMX-induced MASH.

## Materials and methods

### Animals

Adult female Wistar rats (160–180 g) were obtained from the National Cancer Institute’s animal housing (Cairo, Egypt). Rats were kept in a regular environment (24 ± 2ºC, 12-h light–dark cycle, and 40–70% humidity). Rats were housed (5 rats/cage) for one week before the experiment. They were given water ad libitum and free licenses to food. The approval protocols for the study were supported by the ethics committee of the Faculty of Pharmacy, Helwan University, Egypt (Protocol Number: 14 A2023), following the guide for the care and Use of Laboratory Animals published by the US National Institutes of Health (NIH publication No. 85–23 revised 2011).

### Drugs and chemicals

TAMX (Nolvadex ®) was obtained from AstraZeneca (Cairo, Egypt), and BA, along with all other chemicals, was sourced from Sigma-Aldrich Chemical Co (St. Louis, MO, USA).

### Experimental design

Twenty-four adult female rats were randomly allocated into four groups (*n* = 6):

- Control group (vehicle group): Rats were given Tween 80 (1%) orally by gavage once daily for 15 consecutive days.
- TAMX 45 mg/kg group: Rats were given orally 45 mg/ kg of TAMX once daily for 15 consecutive days to induce steatohepatitis.
- TAMX + BA 15 mg/kg group: Rats were given 15 mg/kg of BA. One hour later, 45 mg/kg of TAMX was given. This was administered orally once daily for 15 consecutive days.
- TAMX + BA 30 mg/kg group: Rats were given 30 mg/kg of BA. One hour later, 45 mg/kg of TAMX was given. This was administered orally by gavage once daily for 15 consecutive days.

BA and TAMX solutions were prepared daily by suspending saline containing 1% tween 80 solutions. The dose of TAMX was selected based on our pilot study. The dose of 30 mg/kg of BA is chosen based on previous studies (Sanjay et al. 2021; Ahmed et al. 2023). Meanwhile, the dose of 15 mg/kg of BA is chosen as it is half the previous dose and is nearly equivalent to the human dose (using the body surface area (BSA) normalization method).

### Blood and tissue collection

Twenty-four hours after the last dose of BA, blood samples were acquired from the retro-orbital area and were allowed to clot. The serum was obtained by centrifugation at 3,000 rpm for 20 min and used to assess liver enzymes and lipid profiles. The rats were then euthanized after anesthesia with ketamine (100 mg/kg), and their livers were quickly dissected and weighed. Liver samples were kept in formalin (10%) for histopathological inspections, and other samples were homogenized in ice-cold phosphate-buffered saline, then centrifuged at 3,000 rpm for 30 min and kept at −80°C for biochemical analysis.

### Estimation of relative liver weight according to the equation

$$\mathrm{Relative}\;\mathrm{liver}\;\mathrm{weight}\;=\;\frac{\mathrm{liver}\;\mathrm{weight}\;(\mathrm g)}{\mathrm{Body}\;\mathrm{weight}\;(\mathrm g)}$$

### Estimation of liver enzymes

Serum levels of alanine transaminase (ALT) and aspartate aminotransferase (AST) were determined using colorimetric kits purchased from Diamond Diagnostics (Cat# EC2.6.1.2 and EC2.6.1.1, respectively). The assay is based on the method of Reitman and Frankel (Reitman and Frankel 1957).

### Estimation of lipid profile

Total cholesterol (TC) was estimated spectrophotometrically using EnzyChrom™ AF Cholesterol Assay Kit (Cat# E2CH-100, Hayward, CA, USA). Triglyceride (TG) and high-density lipoprotein cholesterol (HDL-C) were estimated spectrophotometrically using BioMed diagnostic kits (Cat# TG117090 and CKM108025, OR, USA, respectively). Low-density lipoprotein cholesterol (LDL-C) level was calculated using the following formula (Friedewald et al. 1972).

$$\text{LDL}-\text{C}=\text{TC}- (\text{TG}/5+\text{ HDL}-\text{C})$$

### Estimation of oxidative stress markers

Malondialdehyde (MDA) and superoxide dismutase (SOD) in the liver tissue homogenates were estimated spectrophotometrically using the commercial kits from biodiagnostic (Cat# MD 2529 and SOD 2521, Giza, Egypt, respectively).

### Estimation of inflammatory markers

The liver homogenate contents of TNF-α and NF-κB/p65 were measured using the TNF-α Biolegend ELISA kit (Cat# 438204, San Diego, California, USA) and NF-κB/p65 Elabscience ELISA kit (Cat# E-EL-R0674, Texas, USA). All procedures followed the manufacturer’s protocols.

### Estimation of p-AMPK and ACL

The levels of p-AMPK and ACL were estimated using ELISA kits (Fine Biotech, Cat# ER0730, Wuhan, China) and (Antibodies.com, Cat# A79867, Cambridge, UK), respectively.

### Gene expression and quantitative real-time RT-PCR for SREBP1-C and FAS

The total RNA extraction from the liver tissue homogenates was done using an extraction kit (NucleoSpin^®^ REF.740901.250, Macherey- Nagel GmbH, Germany). Following the extraction, a master mix for RT-PCR was applied to the samples (Bioline, SensiFAST™ SYBR® Hi-ROX One-Step Kit, Cat# PI-50217 V, London, UK). The formulated reaction mix samples were utilized in real-time PCR (StepOne Applied Biosystem, USA). After the run, the data obtained were represented in Cycle threshold (Ct). The sequence of primers of the assessed gene (SREBP-1c & FAS*)* and the housekeeping (reference) gene GAPDH are listed in Table 1.

**Table 1:** The sequence of primers utilized for real-time PCR analysis

Gene symbol	Primer sequence from 5′- 3′
SREBP-1c	F: AGCACAGCAACCAGAAACTCAA R: AGGTCTTTCAGTGATTTGCTTTTGT AF286470.2
*FAS*	F: CAAGGGACTGATAGCATCTTTGAGG R: TGTGAAAGTCCTGAACCCTAAGG NM_139194.3
*GAPDH*	F: CACCCTGTTGCTGTAGCCATATTC R: GACATCAAGAAGGTGGTGAAGCAG NM_001394060.2

### Estimation of ACC and CPT-1 by western blot analysis

The liver tissues’ total proteins were extracted via The Ready Prep ™ protein extraction kit (Bio-Rad Inc., Cat#163–2086, Hercules, California, USA) and then quantified via Bradford Protein Assay Kit (SK3041) (Bio Basic Inc., Markham Ontario L3R 8T4 Canada). The separated proteins were transferred to the PVDF membrane and then incubated with the primary antibodies of ACC and CPT-1 (ProteinTech, Cat#15,184–1-AP, Manchester, UK; Santa Cruz Biotechnology Inc., Cat#sc-137104, Dallas, Texas, USA, respectively**)** at 4◦C overnight. The membranes were rinsed in tris-buffered saline with Tween 20 (TBST) buffer, incubated in HRP secondary antibodies solution at room temperature, and then washed in TBST buffer. Clarity™ Western ECL substrate as a chemiluminescent substance (Bio-Rad, Cat#170–5060) was added, and the signals were taken by a CCD camera-based imager. Image analysis was done using ChemiDoc MP imager and normalized by the housekeeping β-actin protein.

### Histopathological examination

The liver tissue samples were fixed in formal saline (10%) for 48 h, washed, dehydrated in a serial dilution of alcohol, and then implanted in paraffin wax media. Via rotary microtome, serial 4–5 μm thick slices were stained with hematoxylin and eosin (H&E) for microscopical inspection. The score of hepatic steatosis for MASLD, which evaluates steatosis, inflammation, and ballooning degeneration, was assessed following the guidelines established by Kleiner et al. (Kleiner et al. 2005).

### Statistical analysis

The data was reported as the mean ± standard error of the mean (SEM). The statistical assessment among groups was conducted using ANOVA, followed by Tukey’s test. *p*-value < 0.05 was considered for statistical significance. The statistical analysis and graphical display of the results were done using GraphPad Prism (Version 8, GraphPad Software, Inc., USA).

## Results

### Effect of BA on the body weight, liver weight, and liver/body weight ratio on TAMX-induced steatohepatitis in female rats

On the last day of the experiment, the body weight of the TAMX-treated rats decreased significantly by approximately 8.82% (*P* < 0.05) compared to the control rats. Concurrent administration of BA (15 and 30 mg/kg) with TAMX showed no marked difference in body weight compared to the TAMX group. Notably, the results revealed no significant variations in liver weight across all groups. However, the liver/body weight ratio in the TAMX group markedly (*P* < 0.05) increased by approximately 11.76% compared to the control group, while concurrent treatment of BA (15 and 30 mg/kg) with TAMX markedly (*P* < 0.05) decreased the ratio by approximately 11.24% and 8.61% respectively compared to the TAMX group (Table 2).

**Table 2:** Effect of BA on the body weight, liver weight, and liver/body weight ratio on TAMX-induced steatohepatitis in female rats

Groups Parameters	Control	TAMX 45 mg/kg	TAMX + BA 15 mg/kg	TAMX + BA 30 mg/kg
Body weight (g)	172.20 ± 3.69	157.00 ± 1.75^#^	166.30 ± 4.05	168.50 ± 0.76^*^
Liver weight (g)	6.51 ± 0.12	6.56 ± 0.11	6.24 ± 0.25	6.45 ± 0.08
Liver/Body weight ratio (g/g)	3.74 ± 0.05	4.18 ± 0.10^#^	3.71 ± 0.10^*^	3.82 ± 0.04^*^

Data are represented as M ± S.E.M (*n *= 6). #: Significant from the control group (*p* < 0.05) , *: Significant from the TAMX group (*p* < 0.05) . TAMX: Tamoxifen, BA: Bempedoic acid

### Effect of BA on liver enzymes in TAMX-induced steatohepatitis in female rats

Rats treated with TAMX markedly increased the serum levels of ALT and AST by twofold (*P* < 0.05) compared to the normal rats. Conversely, the combined treatment of BA (15 and 30 mg/kg) alongside TAMX significantly declined ALT level by 43.75% and 51.77%, respectively, and AST level by 34.39% and 42.3%, (*P* < 0.05), respectively, as compared to the TAMX group (Table 3).

**Table 3:** Effect of BA on liver enzymes and lipid profile in TAMX-induced steatohepatitis in female rats

Groups Parameters	Control	TAMX 45 mg/kg	TAMX + BA 15 mg/kg	TAMX + BA 30 mg/kg
ALT (U/L)	61.17 ± 6.84	137.20 ± 9.22^#^	77.17 ± 8.26^*^	66.17 ± 4.81^*^
AST (U/L)	120.50 ± 12.36	235.20 ± 8.38^#^	154.30 ± 13.83^*^	135.70 ± 10.92^*^
TC (mg/dL)	73.00 ± 5.65	122.20 ± 3.32^#^	86.83 ± 5.19^*^	79.17 ± 4.37^*^
TG (mg/dL)	77.67 ± 5.94	141.20 ± 4.07^#^	95.50 ± 9.44^*^	83.33 ± 5.32^*^
HDL (mg/dL)	52.17 ± 3.34	24.00 ± 0.73^#^	33.83 ± 0.47^#*^	40.17 ± 0.40^#*^
LDL (mg/dL)	5.33 ± 1.08	34.50 ± 1.05^#^	16.17 ± 1.30^#*^	8.83 ± 0.47^*$^

Data are represented as M ± S.E.M (*n* = 6). #: Significant from the control group (*p* < 0.05) , *: Significant from the TAMX group (*p* < 0.05) , $: Significant from the TAMX + BA 15mg/kg group (*p* < 0.05).  TAMX: Tamoxifen, BA: Bempedoic acid, ALT: Alanine transaminases, AST: Aspartate aminotransferase, TC: Total cholesterol, TG: Triglycerides, HDL: High-density lipoprotein, LDL: Low-density lipoproteins

### Effect of BA on lipid profile in TAMX-induced steatohepatitis in female rats

As represented in Table 3, Rats injected with TAMX (45 mg/kg) showed a marked elevation in TG, TC, and LDL by 82%, 67%, and sixfold (*P* < 0.05) compared to the control rats. Additionally, there was a significant decline in HDL by 54% (*P* < 0.05) compared to the control rats. Co-treatment of BA (15 and 30 mg/kg) with TAMX markedly (*P* < 0.05) reduced the elevation in TG serum level by 32% and 41%, respectively. It also significantly (*P* < 0.05) decreased TC by 29% and 35%, respectively. Moreover, LDL levels significantly decreased (*P* < 0.05) following treatment with both doses of BA combined with TAMX, showing reductions of 53% and 74%, respectively. The combination of BA (30 mg/kg) with TAMX markedly reduced (*P* < 0.05) LDL serum level by 45% compared to the combination of BA at a dose of 15 mg/kg with TAMX. Finally, HDL was markedly enhanced (*P* < 0.05) with the concurrent administration of BA (15 and 30 mg/kg) alongside TAMX by 41% and 67%, respectively, compared to the TAMX group.

### Effect of BA on oxidative stress biological markers in TAMX-induced steatohepatitis in female rats

**Fig. 1:**
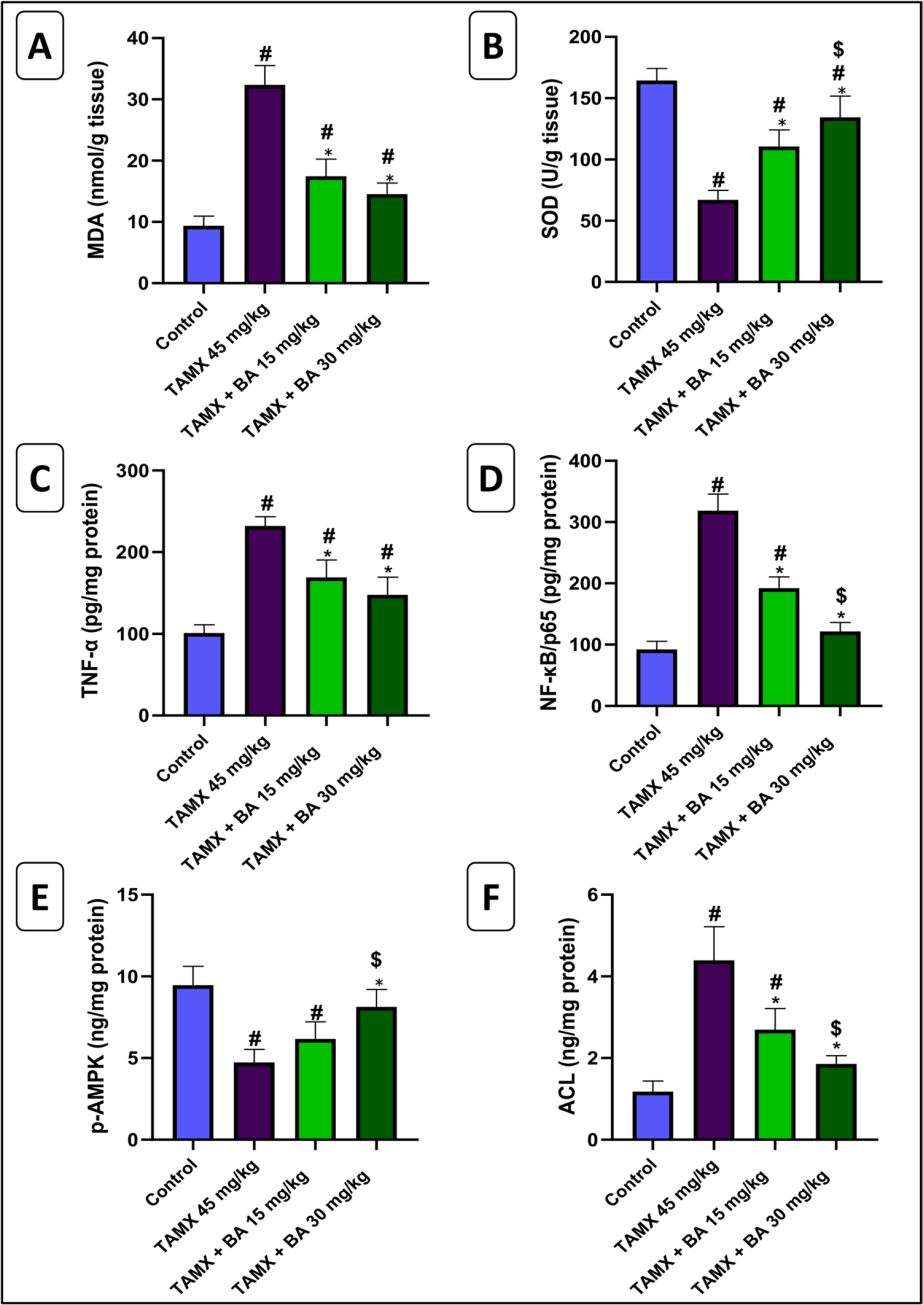
Effect of BA on **A**) MDA, **B**) SOD, **C**) NF-κB/p65, **D**) TNF-α, **E**) p-AMPK, and **F**) ACL in TAMX-induced steatohepatitis in female rats. Data are represented as M ± S.E.M (*n* = 6). #: Significant from the control group (*p* < 0.05), *: Significant from the TAMX group (*p* < 0.05), $: Significant from the TAMX + BA 15mg/kg group (*p* < 0.05). TAMX: Tamoxifen, BA: Bempedoic acid, MDA: Malondialdehyde, SOD: Superoxide dismutase, NF-κB/p65: phosphorylated nuclear factor-kappa B, TNF-α: Tumor necrosis factor-alpha, AMPK: Phosphorylated AMP-activated protein kinase, ACL: ATP-citrate lyase

TAMX (45 mg/kg) significantly (*P* < 0.05) declined SOD activity by 59% and markedly (*P* < 0.05) increased MDA level to about 3.5 fold compared to the control group. Concurrent treatment of BA at a dosage of 15 and 30 mg/kg with TAMX markedly (*P* < 0.05) raised SOD activity by 65% and 100%, respectively, and significantly lowered MDA level by 46% and 55%, respectively when compared to the TAMX group. Interestingly, the administration of BA at 30 mg/kg alongside TAMX markedly (*P* < 0.05) increased SOD activity by 17% compared to the administration of BA at 15 mg/kg with TAMX (Fig. 1A and B).

**Fig. 2:**
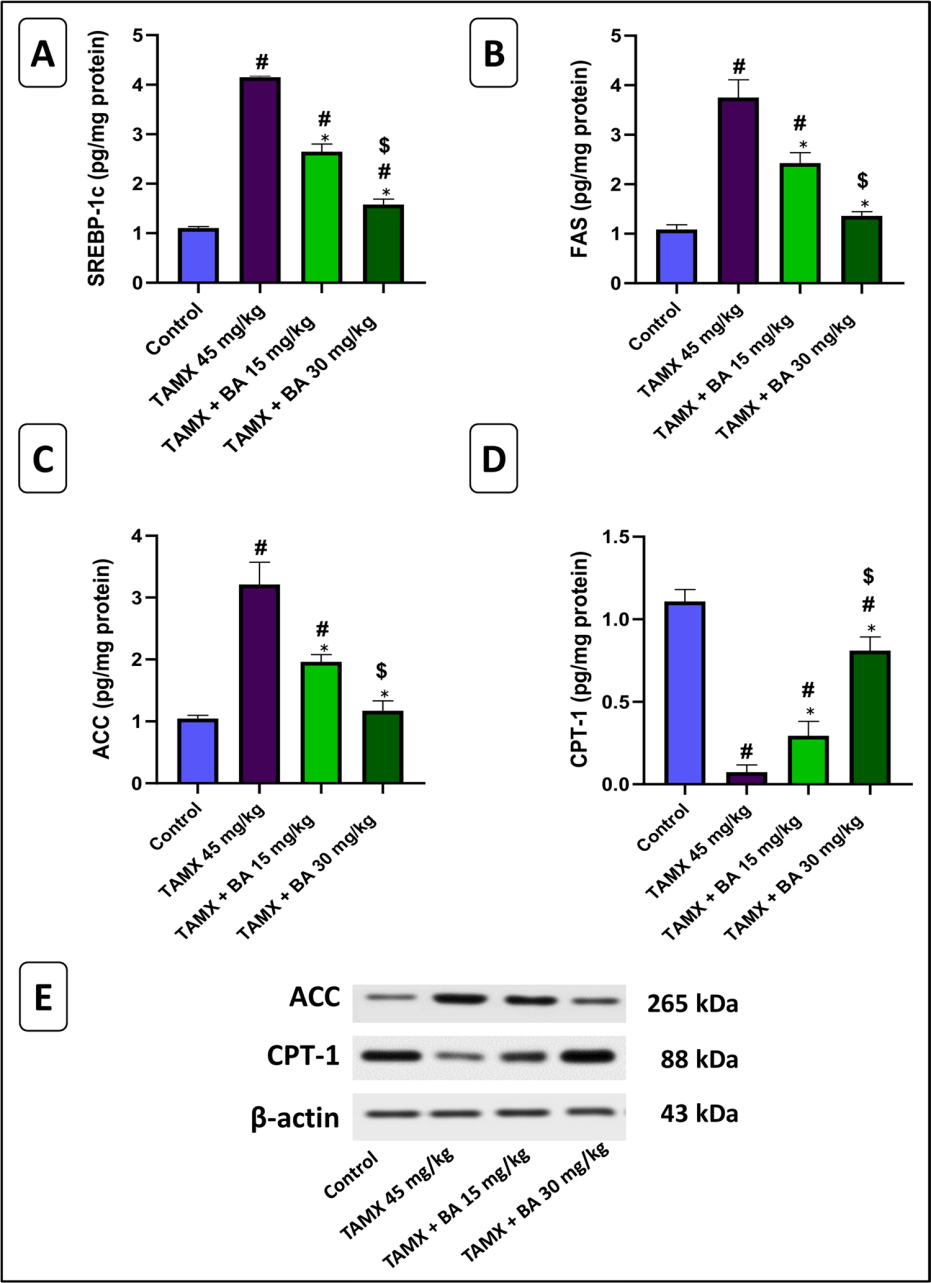
Effect of BA on mRNA relative expression of **A**) SREBP1-C and **B**) FAS, and the level of **C**) ACC level and **D**) the protein expression of CPT-1 in TAMX-induced steatohepatitis in female rats. **E**) Western-blot autoradiography for CPT-1. Data are represented as M ± S.E.M (*n* = 6). #: Significant from the control group (*p* < 0.05), *: Significant from the TAMX group (*p* < 0.05), $: Significant from the TAMX + BA 15mg/kg group (*p* < 0.05). TAMX: Tamoxifen, BA: Bempedoic acid, SREBP-1c: Sterol regulatory element binding protein 1c, FAS: Fatty acid synthetase, ACC: Acetyl-coenzyme A carboxylases, CPT-1: Carnitine palmitoyl transferase 1

### Effect of BA on the inflammatory markers in TAMX-induced steatohepatitis in female rats

Rats administrated with TAMX (45 mg/kg) exhibited a marked increase in NF-κB/p65 and TNF-α levels, approximately 3.5 and 2.3 fold (*P* < 0.05), respectively, compared to the control rats. Conversely, Co-treatment of BA (15 and 30 mg/kg) with TAMX markedly (*P* < 0.05) decreased NF-κB/p65 level by 40% and 62%, respectively, and a significant reduction (*P* < 0.05) in TNF-α level by 27% and 36%, respectively, compared to the TAMX group. Moreover, a marked (*P* < 0.05) decline in the level of NF-κB/p65 by 37% was observed in the TAMX + BA 30 mg/kg group compared to the TAMX + BA 15 mg/kg group (Fig. 1C and D).

### Effect of BA on p-AMPK level and ACL activity in TAMX-induced steatohepatitis in female rats

As represented in (Fig. 1E and F), rats administered with TAMX demonstrated a marked reduction in p-AMPK level and a marked enhancement in ACL activity of approximately 50% and 3.7 folds, respectively (*P* < 0.05) compared to the control group. In contrast, the concurrent administration of BA (15 and 30 mg/kg) with TAMX significantly enhanced p-AMPK level by approximately 30.23% and 71.88%, respectively (*P* < 0.05), while also reducing ACL activity by approximately 38.72% and 57.85%, respectively, compared to the TAMX-treated group. Interestingly, treatment of the high dose of BA (30 mg/kg) together with TAMX resulted in a marked increase in p-AMPK level by approximately 31.99% (*P* < 0.05) and a marked decrease in ACL activity by approximately 31.05% (*P* < 0.05) compared to the oral treatment of a low dose of BA (15 mg/kg) in combination with TAMX.

### Effect of BA on mRNA relative expression of SREBP1-C and FAS in TAMX-induced steatohepatitis in female rats

As demonstrated in Fig. 2A and B), TAMX markedly enhanced the gene expression of SREBP-1C and FAS by approximately 3.7 fold and 3.4 fold, respectively (*P* < 0.05), compared to the control rats. While parallel treatment of BA (15 and 30 mg/kg) with TAMX significantly reduced the gene expression of SREBP-1C by 36% and 61.97%, respectively (*P* < 0.05), and markedly decreased the expression of its target gene FAS by 35% and 63.76%, respectively, compared to the TAMX group. Interestingly, the co-treatment of BA at 30 mg/kg with TAMX showed a marked decline in the gene expression of SREBP-1C and FAS by 40.32% and 43.96%, respectively, when compared to the TAMX + BA 15 mg/kg group. 

### Effect of BA on ACC level and CPT-1 expression in TAMX-induced steatohepatitis in female rats

Our results indicated that TAMX significantly increased hepatic ACC protein expression by approximately 3.46 fold (*P* < 0.05), and significantly (*P* < 0.05), decreased hepatic CPT-1 protein expression by 93%, in comparison with the control group. BA co-treatment with TAMX (15 and 30 mg/kg) significantly reduced ACC protein expression by 38.86% and 63.56%, respectively (*P* < 0.05), compared to the TAMX group. Additionally, it markedly increased (*P* < 0.05) CPT-1 protein expression by 75% and 91%, respectively, compared to the TAMX group. Importantly, the synchronized administration of BA (30 mg/kg) with TAMX markedly (*P* < 0.05) increased CPT-1 expression by 64% and decreased ACC expression by 40% compared to the synchronized administration of BA (15 mg/kg) with TAMX. (Fig. 2C,D and F).

### Effect of BA on liver histopathological changes in TAMX-induced steatohepatitis in female rats

**Fig. 3:**
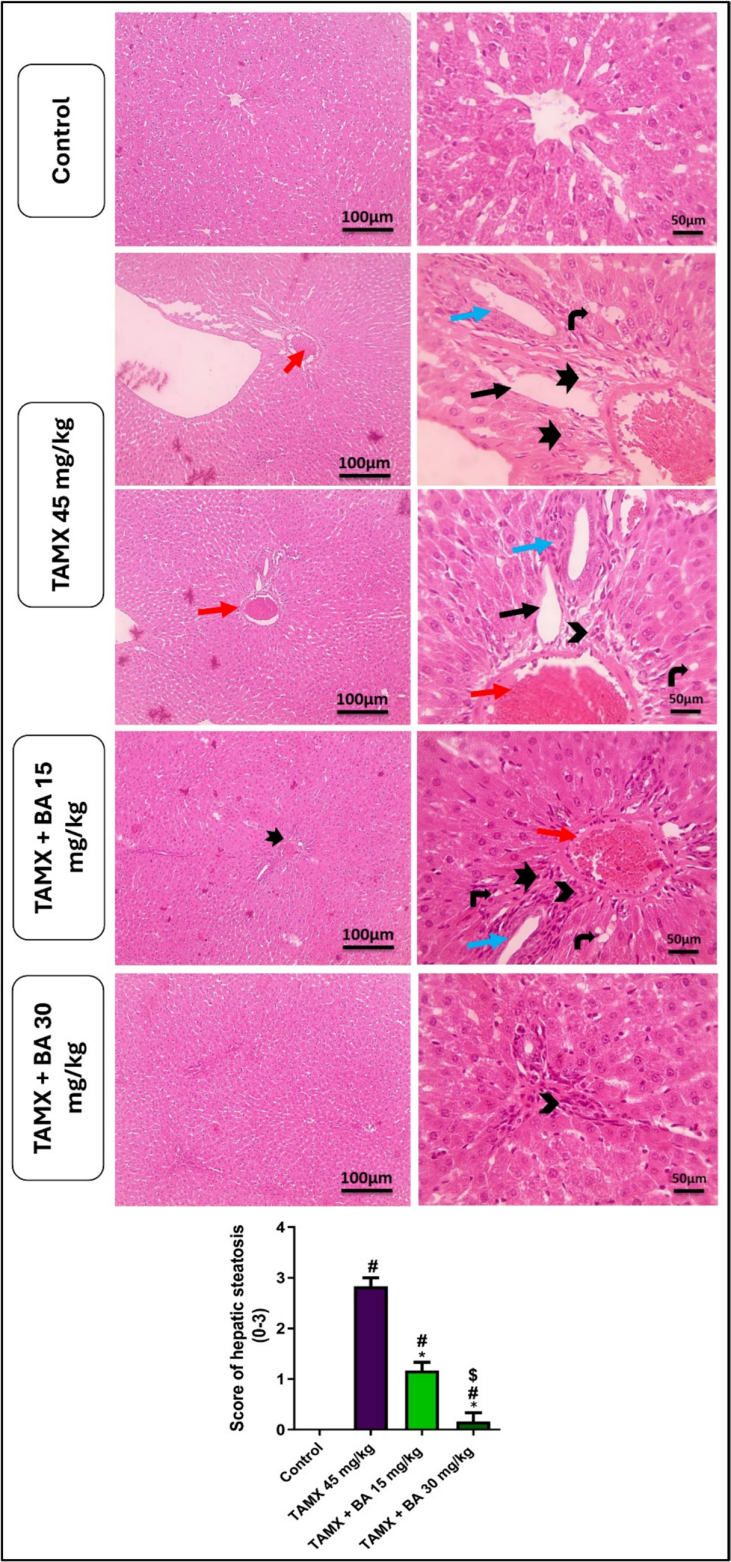
Effect of BA on liver histopathological changes in TAMX-induced steatohepatitis in female rats. Liver sections show a normal arrangement of hepatocytes around central veins with normal sinusoids in the control group. Liver sections from (TAMX 45 mg/kg) group showing extension of portal areas due to vascular dilation (*) and congestion (red arrow), dilated lymphatics (thin black arrow), dilated bile ductules (thin blue arrow), fibrosis (thick black arrow), bile ductules proliferation and mononuclear cells infiltration (arrowheads), besides vacuolization in hepatocytes (angled black arrow). Liver sections from the TAMX + BA 15 mg/kg group showed similar lesions with a slight extent. Liver sections from the TAMX + BA 30 mg/kg group showed very few mononuclear cell infiltrations (arrowhead). Magnifications × : 100 bar 100 and × : 400 bar 50. Data are represented as M ± S.E.M (*n* = 6). #: Significant from the control group (*p* < 0.05), *: Significant from the TAMX group (*p* < 0.05), $: Significant from the TAMX + BA 15mg/kg group (*p* < 0.05). TAMX: Tamoxifen, BA: Bempedoic acid

The histological findings of the liver sections from the control group demonstrated a well-organized histological structure. Meanwhile, Liver sections of the TAMX group displayed focal hepatic necrosis linked to inflammatory cell infiltration, sinusoidal leukocytosis, lipid droplets in hepatocytes, congestion of the central vein, and oval cell proliferation. The concurrent administration of BA with TAMX improved the histopathological findings associated with TAMX alone in a dose-dependent manner, as presented in Fig. 3. The TAMX-treated rats showed a significant (*P* < 0.05) increase in NAS score compared to the control rats. Treatment with BA (15 and 30 mg/kg) significantly reduced NAS score by (*P* < 0.05), compared to the TAMX group.

## Discussion

Based on our understanding, this is the first study to demonstrate the hepatoprotective of BA against TAMX-induced steatohepatitis in rats. TAMX is an estrogen antagonist that is widely used as a therapeutic agent for breast cancer. However, the adverse effects associated with the use of TAMX lead to low patient adherence, discontinuation of the drug, and, ultimately, unexpected clinical outcomes (Condorelli and Vaz-Luis 2018). Fatty liver and steatohepatitis are the most commonly reported side effects associated with TAMX use in humans (Lee et al. 2020) and experimental animals (Leal et al. 2018; Hammad et al. 2018). Recently, there has been an urgent requirement to discover a novel therapeutic strategy for overcoming steatohepatitis induced by TAMX. Therefore, the current study aimed to inspect the possible hepatoprotective effect of BA, as a novel hypolipidemic agent, against TAMX-induced steatohepatitis. BA has been previously shown to exert protective effects against metabolic dysfunctions by modulating lipid metabolism and inflammatory responses (Biolo et al. 2022).

In the current study, oral treatment of TAMX at a dose of 45 mg/kg in rats induced liver damage accompanied by steatohepatitis, as evidenced by a significant increase in the levels of liver enzymes such as ALT and AST, consistent with findings from Kamel et al. (Kamel and Elariny 2023). Due to cellular injury and loss of cell integrity caused by TAMX, cytoplasmic liver enzymes are released into the systemic circulation. Significant improvement in liver injury was achieved with BA co-treatment, suggesting its potential to inhibit one or more signaling pathways involved in liver injury.

Furthermore, TAMX increases serum levels of TG, TC, and LDL while decreasing serum HDL, confirming the occurrence of TAMX-induced hyperlipidemia in agreement with the findings of El-Asfar et al. (El-Asfar et al. 2021). Estrogen plays an essential role in regulating lipid metabolism in the liver, and since TAMX is an estrogen antagonist, it leads to increased levels of LDL cholesterol and triglycerides (Condorelli and Vaz 2018). On the bright side, co-administration of BA resulted in a marked improvement in the lipid profile. BA stimulates an increase in membrane receptors for LDL, thus enhancing its clearance from the bloodstream (Biolo et al. 2022).

Several studies linked the hepatotoxic effects of TAMX with the overproduction of ROS and inflammatory responses (Famurewa et al. 2020; Yu et al. 2020). Our results revealed that TAMX boosted oxidative stress by a marked elevation in MDA level and a significant decrease in SOD activity. TAMX is known to disrupt mitochondrial respiratory chain activity in the liver, resulting in the elevation of ROS (Massart et al. 2013; Satapathy et al. 2015). TAMX-induced mitochondrial dysfunction severely compromised the antioxidant defense mechanism and promoted the overproduction of ROS. Consequently, polyunsaturated fatty acids underwent oxidation, generating MDA, which formed adducts with proteins and DNA bases. Furthermore, the reduction in hepatic SOD activity intensified ROS accumulation and accelerated lipid peroxidation.

ROS induces fatty acid peroxidation, which subsequently triggers inflammation and fibrogenesis through the activation of Kupffer and Ito cells. This initiates a vicious cycle, resulting in increased production of ROS (Miele et al. 2017). It is well-documented that BA is classified as an AMPK activator (Biolo et al. 2022). Additionally, it was reported that phosphorylation of AMPK attenuated the oxidative stress, suppressed the production of ROS, and boosted the enzymatic antioxidant activity (Wu et al. 2014). Our study documented the antioxidant activity of BA, as evidenced by a significant reduction in MDA level and a notable increase in SOD level. Cytokines, primarily produced by activated leukocytes, are central components of the inflammatory cascade. The current study revealed heightened inflammatory responses, as indicated by increased TNF-α and NF-κB/p65 levels in the TAMX group. This was in harmony with Abd El-Haleim and Sallam. (Abd El-Haleim and Sallam 2022). TNF-α is considered a key member of the inflammatory cytokine family, playing a crucial role in initiating and driving nearly all aspects of the systemic inflammatory response syndrome (Dinarello et al. 1993). It can activate NF-κB, which subsequently enhances the release of further inflammatory cytokines. On the contrary, co-administration of BA curbed the hepatic TNF-α and NF-κB levels. This can be attributed to the antioxidant properties of BA, which scavenge ROS and consequently reduce the production of pro-inflammatory cytokines. Moreover, BA is an AMPK activator; therefore, it suppresses the inflammatory responses against TAMX-induced steatohepatitis. Many studies demonstrated that AMPK activation declines the expression of proinflammatory cytokines and consequently attenuates inflammation (Salminen et al. 2011; Huang et al. 2015). Our findings align with previous reports indicating that AMPK activation attenuates hepatic steatosis and oxidative stress, supporting the role of BA in MASH management (Hammad et al. 2023; Bekheit et al.. 2025).

In line with the findings mentioned above, histopathological examinations confirmed TAMX-induced steatohepatitis, as evidenced by hepatic necrotic lesions associated with mononuclear inflammatory infiltrates, vacuolation in hepatocytes, portal vein congestion, and lipid droplets within hepatocytes. Co-administration of BA showed, in a dose-dependent manner, significant hepatoprotective effects against TAMX-induced fatty liver in rats. Treatment with BA alleviated oxidative stress and inflammation, which is linked with the lower levels of AST and ALT, lipid profile amendment, and improved hepatocyte architecture (Biolo et al. 2022).

Many studies documented the implication of TAMX in the development of MASLD (Kim et al. 2018; Yoo et al. 2020)**.** MASLD is developed via the accumulation of TGs in the hepatocytes, which are created by the esterification of FFA and glycerol. SREBP-1c is the main isoform of SREBPs family that regulates the synthesis of fatty acids in the liver (Pettinelli et al. 2011)**.** SREBP-1c enhances gene transcription of lipogenesis, including ACL, ACC, and FAS (Zhao et al. 2014). Our results showed that TAMX promoted lipogenesis in the liver, as indicated by a significant increase in the transcription factor SREBP-1c and FAS gene expression, accompanied with an increase in ACL activity and ACC expression. Moreover, TAMX significantly reduced p-AMPK level, leading to further activation of ACC and subsequently decreasing CPT-1 expression, which increased lipogenesis, lipid accumulation, and inflammation, consistent with findings from other studies (Cole et al. 2010; Ismail et al. 2020). In contrast, co-treatment with BA significantly, in a dose-dependent manner, increased the p-AMPK level and CPT-1 expression while reducing SREBP-1c and FAS expression, along with ACL activity and ACC expression, compared to the TAMX group. As an AMPK activator, BA plays a vital role in regulating energy metabolism. Activated p-AMPK suppresses SREBP-1c expression and increases ACC phosphorylation, resulting in its inactivation. Since ACL catalyzes the ATP-dependent conversion of citrate to ACC, it serves as the precursor for fatty acid and cholesterol synthesis (Biolo et al. 2022). Therefore, the inhibition of ACL activity by BA suppresses lipogenesis, lipid accumulation, and inflammation as represented in our study. Our findings align with earlier studies demonstrating that AMPK activation reduces lipogenesis, inflammation, and oxidative stress while enhancing mitochondrial fatty acid β-oxidation, thereby slowing the progression of MASH (Bekheit et al. 2025).

CPT-1 is the rate-limiting enzyme for mitochondrial β-oxidation in the liver, where it facilitates the import of long-chain fatty acids into the mitochondria for β-oxidation (Sepa-Kishi et al. 2016). Consequently, reduced levels of CPT-1 are associated with the process of lipogenesis, as demonstrated in our study, where TAMX administration significantly decreased CPT-1 levels, leading to reduced β-oxidation and increased fatty acid accumulation in liver cells. On the other hand, the co-administration of BA, in a dose-dependent manner, significantly elevated CPT-1 levels through AMPK activation and ACC inactivation, resulting in increased β-oxidation and reduced fatty acid accumulation in liver cells.

**Fig. 4:**
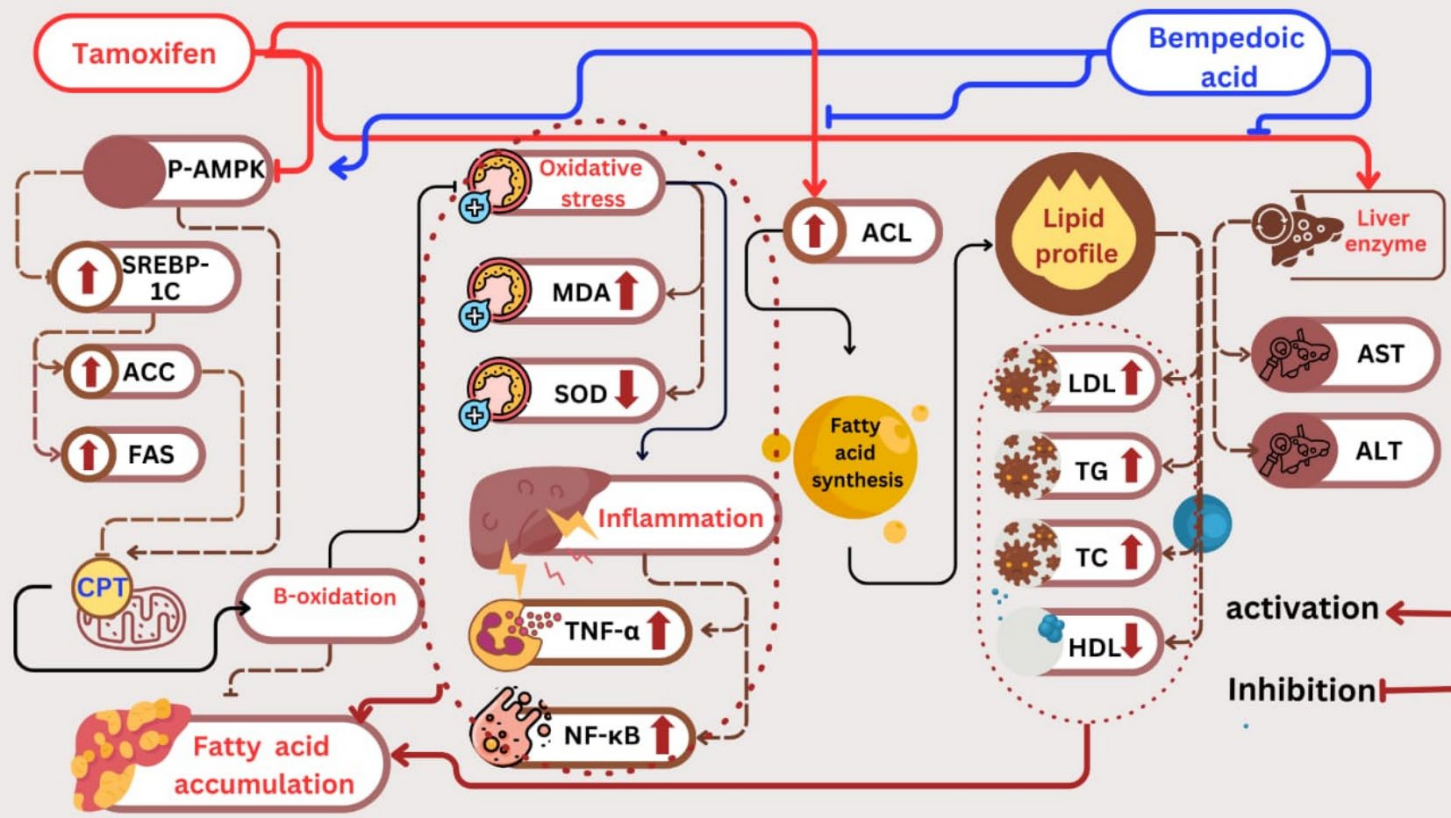
A summary of the protective mechanisms of BA against tamoxifen-induced steatohepatitis in female rats. p-AMPK: phosphorylated AMP-activated protein kinase, SREBP-1C: Sterol regulatory element binding protein −1C, FAS: Fatty acid synthase, ACC: Acetyl-CoA carboxylase, CPT-1: Carnitine palmitoyl transferase I, SOD: Superoxide dismutase, MDA: Malondialdehyde, TNF-α: Tumor necrosis factor alpha, NF-κB/p65: phosphorylated nuclear factor-kappa B, ACL: ATP citrate lyase, TC: total Cholesterol, TG: Triglycerides, HDL: High-density lipoprotein, LDL: Low-density lipoproteins, ALT: Alanine transaminases, AST: Aspartate aminotransferase

Thus, BA exhibited notable antioxidant and anti-inflammatory effects. Its protective effect was achieved through its role as an AMPK activator, which in turn reduces ACL activity and modulates the SREBP-1c/ACC/FAS/CPT-1 pathway. This enhances β-oxidation, suppressing the link between oxidative stress and inflammation, ultimately lowering fatty acid buildup and minimizing cellular injury and steatohepatitis (Fig. 4).

## Conclusion

The current study suggests that BA has protective effects against TAMX-induced steatohepatitis and may have potential as a preventive and supportive therapy. BA attenuated liver tissue damage, improved liver enzymes and lipid profiles, and reduced oxidative stress and inflammation, helping to restore the balance between lipid formation and fatty acid oxidation. Given its lipid-lowering and anti-inflammatory effects, BA could be a promising therapeutic candidate for TAMX-induced liver injury. However, further preclinical and clinical studies are needed to confirm its long-term efficacy and safety.

## Study limitations

- This study was conducted in a rat model, which, while providing valuable mechanistic insights, may not fully replicate human physiology. Differences in metabolism, immune responses, and drug interactions between rodents and humans could influence the translational applicability of the findings. Therefore, further clinical studies are necessary to validate the therapeutic potential of BA in humans.
- This study focused on specific biochemical and histopathological markers of TAMX-induced steatohepatitis and BA’s protective effects. However, other metabolic pathways, such as autophagy, which plays a critical role in lipid metabolism and cellular homeostasis, were not evaluated. Future studies should investigate these pathways to provide a more comprehensive understanding of BA’s mechanisms of action.

## Supplementary Information

Below is the link to the electronic supplementary material.

Supplementary file1 (DOCX 74 KB)

## Data Availability

All source data for this work (or generated in this study) are available upon reasonable request.
