# Supplementary material for: Protective potential of bempedoic acid as an AMPK activator in tamoxifen-induced steatohepatitis in rats
Source: Naunyn Schmiedebergs Arch Pharmacol. 2025 Apr 10;398(9):12661–72. doi: 10.1007/s00210-025-04047-5 (PMC12449389; doi:10.1007/s00210-025-04047-5)
Supplement: Supplementary file 1 — Supplementary file1 (DOCX 74 KB) [file 210_2025_4047_MOESM1_ESM.docx]

**BETA ACTIN**


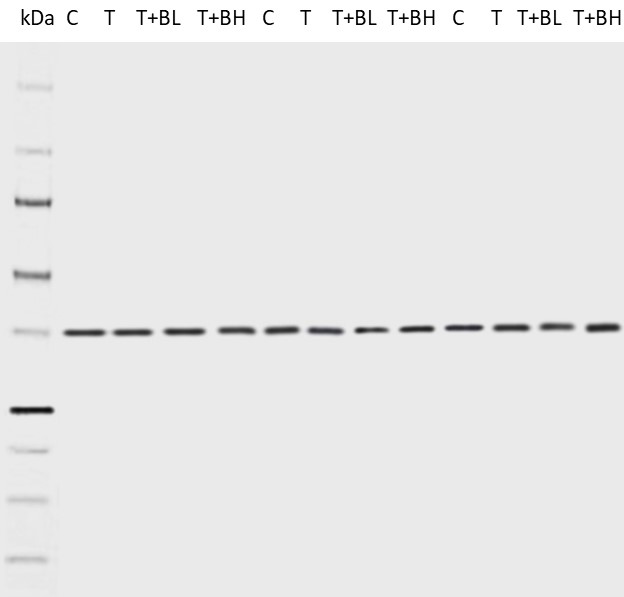


C: control

T: tamoxifen

BL: low dose of Bempedoic acid (15 mg⁄kg)

BH: high dose of Bempedoic acid (15 mg⁄kg)

**ACC**


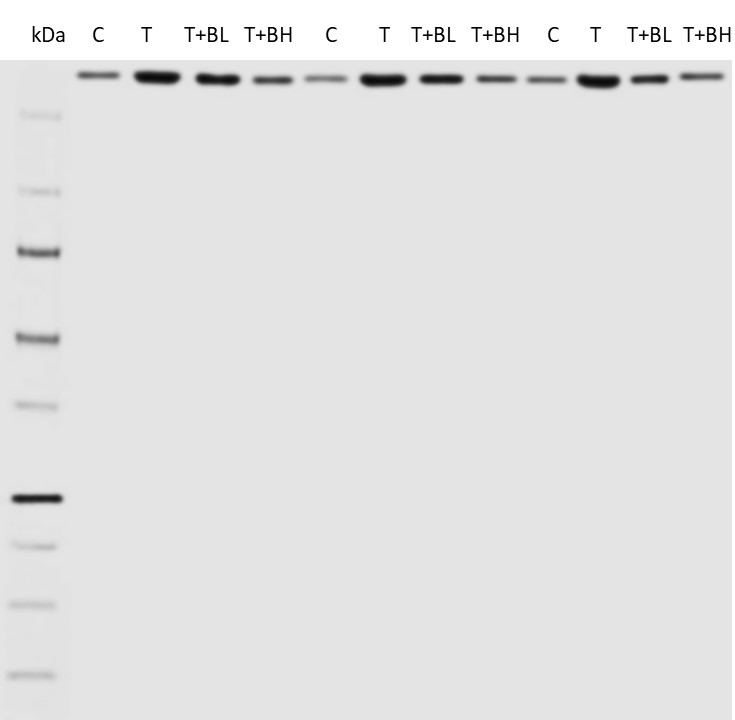


C: control

T: tamoxifen

BL: low dose of Bempedoic acid (15 mg⁄kg)

BH: high dose of Bempedoic acid (15 mg⁄kg)

**CPT-1**


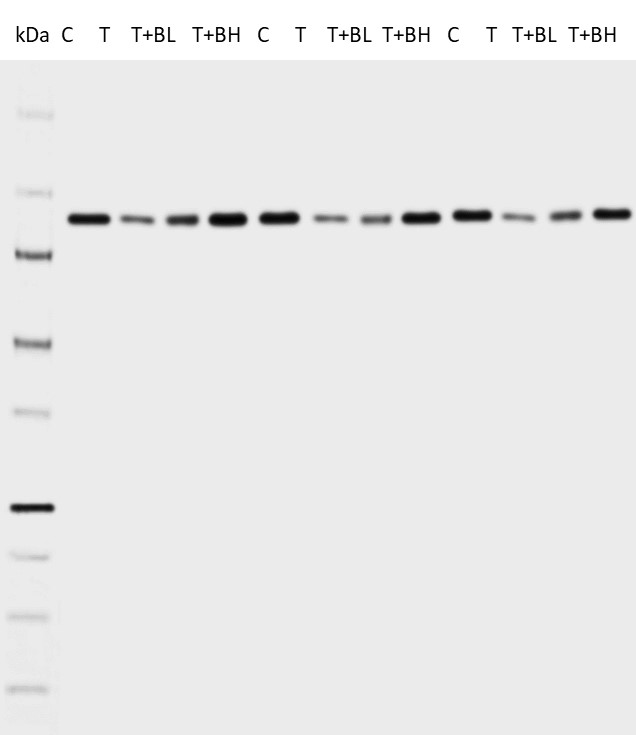


C: control

T: tamoxifen

BL: low dose of Bempedoic acid (15 mg⁄kg)

BH: high dose of Bempedoic acid (15 mg⁄kg)
